# A subunit vaccine based on P97R1, P46, P42, and P65 from *Mycoplasma hyopneumoniae* can induce significant immune response in piglets

**DOI:** 10.3389/fvets.2024.1493650

**Published:** 2024-11-13

**Authors:** Yintao He, Kaiyuan Xie, Zhongmao Yuan, Ting Ouyang, Anran Dong, Bing Ling, Weijun Zeng, Yiqi Fang, Yiwan Song, Lianxiang Wang, Hongxing Ding, Mingqiu Zhao, Shuangqi Fan, Lin Yi, Dongfang Zhao, Jinding Chen

**Affiliations:** ^1^College of Veterinary Medicine, South China Agricultural University, Guangzhou, China; ^2^Key Laboratory of Zoonosis Prevention and Control of Guangdong Province, South China Agricultural University, Guangzhou, China; ^3^Wen’s Group Academy, Wen’s Foodstuffs Group Co., Ltd., Xinxing, China; ^4^Yunfu Branch, Guangdong Laboratory for Lingnan Modern Agriculture, Guangzhou, China

**Keywords:** porcine *Mycoplasma pneumonia*, baculovirus expression system, subunit vaccines, immunity, co-expression of multiple promoters

## Abstract

*Mycoplasma pneumonia* (MPS), caused by *Mycoplasma hyopneumoniae* (Mhp), is a chronic, airborne respiratory disease that poses a significant threat to the global swine industry. The P97 and P46 proteins are major antigens of Mhp, with the R1 region of P97 possessing full adhesive capability. Studies have shown that the main antigenic regions of Mhp P42 and P65 proteins exhibit strong immunogenicity. In this study, we first linked the genes encoding P97R1 and P46 proteins to form the P97R1P65 gene and subsequently constructed three shuttle plasmids: pFBD-P97R1P46, pFBD-P97R1P46-p65, and pFBD-P65-P42. These proteins were expressed using the Bac to Bac system and formulated into subunit vaccines for mouse immunization. Mouse experiments indicated that the P97R1P46 + P65-P42 protein combination elicited higher levels of specific antibodies, IL-2, IL-4, and CD8^+^ T cells compared to other subunit vaccine groups, a finding further validated in subsequent mouse challenge protection experiments. Therefore, we utilized the MultiBac expression system to co-express P97R1P46, P65, and P42 proteins in the pFastMultibacDual vector for immunization experiments in piglets. The piglet immunization experiments demonstrated that the Mhp subunit vaccine prepared in this study could induce specific antibodies against Mhp, with the combination of P97R1P46, P65, and P42 proteins inducing the highest level of humoral immunity. This study provides valuable insights for the development of Mhp subunit vaccines.

## 1 Introduction

*Mycoplasma hyopneumoniae* (Mhp) is the smallest prokaryotic organism found in nature, with a diameter of approximately 0.2 to 0.8 μm. Its structure is simple and consists only of a cell nucleus, ribosomes, and a cell membrane. Owing to the lack of cell walls, they often exhibit pleomorphism. Mhp belongs to the class Mollicutes and the genus Mycoplasma (1). Compared to other types of prokaryotic organisms, the genome of Mhp is relatively small, typically ranging between 850 to 980 kb. Each different type of Mhp contains between 528 to 691 genes encoding proteins. Among these genes, only about 20% are used to encode membrane proteins. However, the function of most of these membrane proteins remains unclear (2). Virulent strains of Mhp possess an approximately 5,000 bp RABD (Repeated Arbitrary DNA Sequence) fragment more than avirulent strains. The virulent strains are capable of inducing more severe pulmonary lesions in affected animals (3, 4).

Currently, the prevention and control of Mhp primarily rely on vaccination and enhanced livestock management practices. Vaccination helps alleviate clinical symptoms and pulmonary lesions in animals affected by Mhp (5–7). Adhesion and colonization of the host by Mhp are critical steps in the pathogenic process. The P97 protein plays a significant role in the adhesion process of Mhp and exhibits strong immunogenicity, making it a commonly used candidate protein for novel subunit vaccines. Among its domains, the R1 region serves as the primary antigenic determinant cluster (8, 9). P46 and P65 are currently among the most extensively studied membrane proteins, demonstrating potential as subunit vaccine candidates (10–14). P42 is a heat shock protein of Mhp, capable of being significantly expressed under stress conditions. Studies have shown that specific antibodies against P42 can inhibit the growth of Mhp (15, 16).

The insect cell expression system is suitable for expressing complex exogenous proteins. It can perform post-translational modifications on exogenous proteins, enabling them to have structures and functions similar to natural proteins. This system offers advantages such as high expression levels and good biological activity of the expressed proteins (17). The Bac-to-Bac system is currently the most widely used insect cell expression system. It allows for the simultaneous expression of two exogenous genes and is suitable for large-scale industrial production (18). In this study, the Bac to Bac system was used to express P46P97R1 individually, co-express P46P97R1 and P65, and co-express P65 and P42. However, research has shown that the Bac to Bac system struggles to co-express more than two viral coat proteins. Addressing this need, Kapil Gupta and their team developed the MultiBac system. The MultiBac system transfer vectors’ expression cassettes have multiple restriction enzyme sites on both sides, allowing for the assembly of multiple expression cassettes. Alternatively, multiple donor plasmids can be fused into a single recipient vector via LoxP sites in a reaction mediated by Cre recombinase. This enables the system to express complex arrays of proteins from a single recombinant baculovirus (19). In simple terms, the MultiBac system enables the co-expression of multiple protein subunits within the same host cell, thus efficiently obtaining multi-protein complexes (20). Therefore, this study conducted the co-expression of recombinant proteins P46P97R1, P65, and P42 was conducted using the MultiBac system.

## 2 Materials and methods

### 2.1 Cell culture and virus

The cells of *Spodoptera frugiperda* (SF9) were cultured in SFM insect medium (Sino Biological) and maintained at 27°C for virus propagation. The cells of High Five (BTI-TN-5B1-4) were cultured in HF insect medium (Sino Biological) and maintained at 27°C for protein expression. The anti-His antibody was purchased from Sino Biological (Beijing, China). The nucleotides encoding P97R1, P46, P65, and P42 proteins of the Mhp 168 strain (GenBank ID: CP002274.1) were optimized for codon usage, synthesized, and cloned into the pFBD or pFBDM vectors. All gene synthesis and insect codon optimization in the study was done by Sangon Biotech (Shanghai, China).

### 2.2 Immunofluorescence assay and western blot analysis

In this experiment, a successfully constructed P3 generation recombinant baculovirus was used to infect SF9 cells at an multiplicity of infection (MOI) of 5. After 72 h of cultivation at 27°C, indirect immunofluorescence analysis was employed to assess the expression of the target protein in insect cells. The cells were fixed with 4% paraformaldehyde, followed by incubation with anti-His mouse monoclonal antibodies as the primary antibody and FITC-conjugated rabbit anti-mouse antibodies for detection. Additionally, the High Five cells were infected with MOI values of 5, 10, and 15, and cell samples were collected at different time points. Anti-His antibodies served as the primary antibody, and HRP-conjugated goat anti-mouse antibodies were used as the secondary antibody for SDS-PAGE and western blot analysis of recombinant protein expression. Protein band detection was performed using the Tanon Fine-do X6 chemiluminescence imaging system. Protein semi-quantification analysis was also conducted using the same method.

### 2.3 Preparation vaccine

The four recombinant baculoviruses constructed in this experiment were individually used to infect High Five cells at the optimal MOI to produce antigens, and the proteins were harvested at the optimal time points. The sonicated cell lysates were centrifuged at 4°C at 11,500 × g for 15 min. According to the manufacturer’s instructions, the expression products of three recombinant baculoviruses (P97R1P46, P97R1P46-P65, P65-P42) were mixed (w/o) with PGA (Pharmgate, Minnesota, American) adjuvant at a volume ratio of 5:1. The expression products of P97R1P46-P65-P42 was mixed (w/o/w) with ISA 201 VG (Seppic, Paris, France) adjuvant at a volume ratio of 1:1.

### 2.4 Animal immunization and challenge experiment

For the mouse immunization and challenge experiments, 42 healthy Kunming mice (Southern Medical University, Guangzhou, China) aged 3 weeks were randomly divided into 6 groups, with 7 mice in each group. Subcutaneous multi-site injections of the prepared Mhp subunit vaccines were administered to the mice. A booster immunization was given at the same dose 2 weeks after the initial immunization. Blood samples were collected via retro-orbital sinus puncture at 7, 14, 21, and 28 days after the initial immunization to isolate mouse serum for antibody and cytokine detection. On day 28 after the initial immunization, 3 randomly selected mice from each group were euthanized under ether anesthesia, and their spleen lymphocytes were isolated for flow cytometric analysis. Two weeks after the booster immunization, the remaining mice in each group were challenged with Mhp Jinan strain freeze-dried tissue virus (5 mL/100 MID) via intraperitoneal injection (150 μL/mouse) and intranasal instillation (50 μL/mouse). Clinical signs were observed post-challenge. At 28 days post-challenge, the mice were euthanized under ether anesthesia, and their lungs were examined to assess the extent of lung lesions using a 28-point scoring system (Madec and Kobisch method). Lung tissues from each group of mice were collected and fixed in the 4% paraformaldehyde solution.

For the pig immunization experiment, 15 healthy piglets aged 1 month, negative for Mhp antibodies, were randomly divided into 5 groups, with 3 piglets in each group. Immunization was performed via intramuscular injection. The initial immunization was administered on day 0, and a booster immunization at the same dose was given on day 14. Blood samples were collected at 7, 14, 21, and 28 days after the initial immunization to isolate serum. The specific antibodies against Mhp and the levels of IFN-γ, IL-2, and IL-4 cytokines were detected using ELISA kits. Clinical symptoms of the immunized pigs were observed daily. The animal experimental procedures were approved by the Experimental Animal Ethics Committee of South China Agricultural University (Approval No. 2021F503).

### 2.5 Flow cytometry

After 28 days post-initial immunization, spleen lymphocytes were isolated from mice and diluted to 10^6^ to 10^8^ cells/mL. Then, 100 μL of the diluted cells were pipet-ted into 1.5 mL opaque EP tubes. Fluorescent-labeled CD3-APC, CD4-FITC, and CD8-PE antibodies were added, and a negative control group (without antibodies), CD3 positive control group, CD4 positive control group, and CD8 positive control group were simultaneously set up. After incubating on ice for 30 min, 400 μL of PBS was added to resuspend the cells. Data acquisition was performed using a flow cytometer, and data analysis was conducted using CytExpert 2.2.

### 2.6 Histopathology and histology research

After 28 days post-infection, mice were anesthetized with ether, dissected, and their lungs were isolated. The isolated mouse lungs were fixed in the 4% paraformaldehyde solution and sent to Wuhan Cervellera Biotechnology (Wuhan, China) for paraffin embedding and sectioning. The sections were stained with hematoxylin and eosin (H&E) and observed under an electron microscope.

### 2.7 Statistical analysis

Data analysis was conducted using GraphPad Prism 6, employing one-way analysis of variance (ANOVA) for inter-group statistical analysis. In the notation, * denotes *p* < 0.05, ** denotes *p* < 0.01, *** denotes *p* < 0.001, **** denotes *p* < 0.0001, and “ns” indicates *p* > 0.05, signifying no significant difference.

## 3 Results

### 3.1 Gene design and construction of shuttle plasmids for recombinant proteins

**Figure 1:**
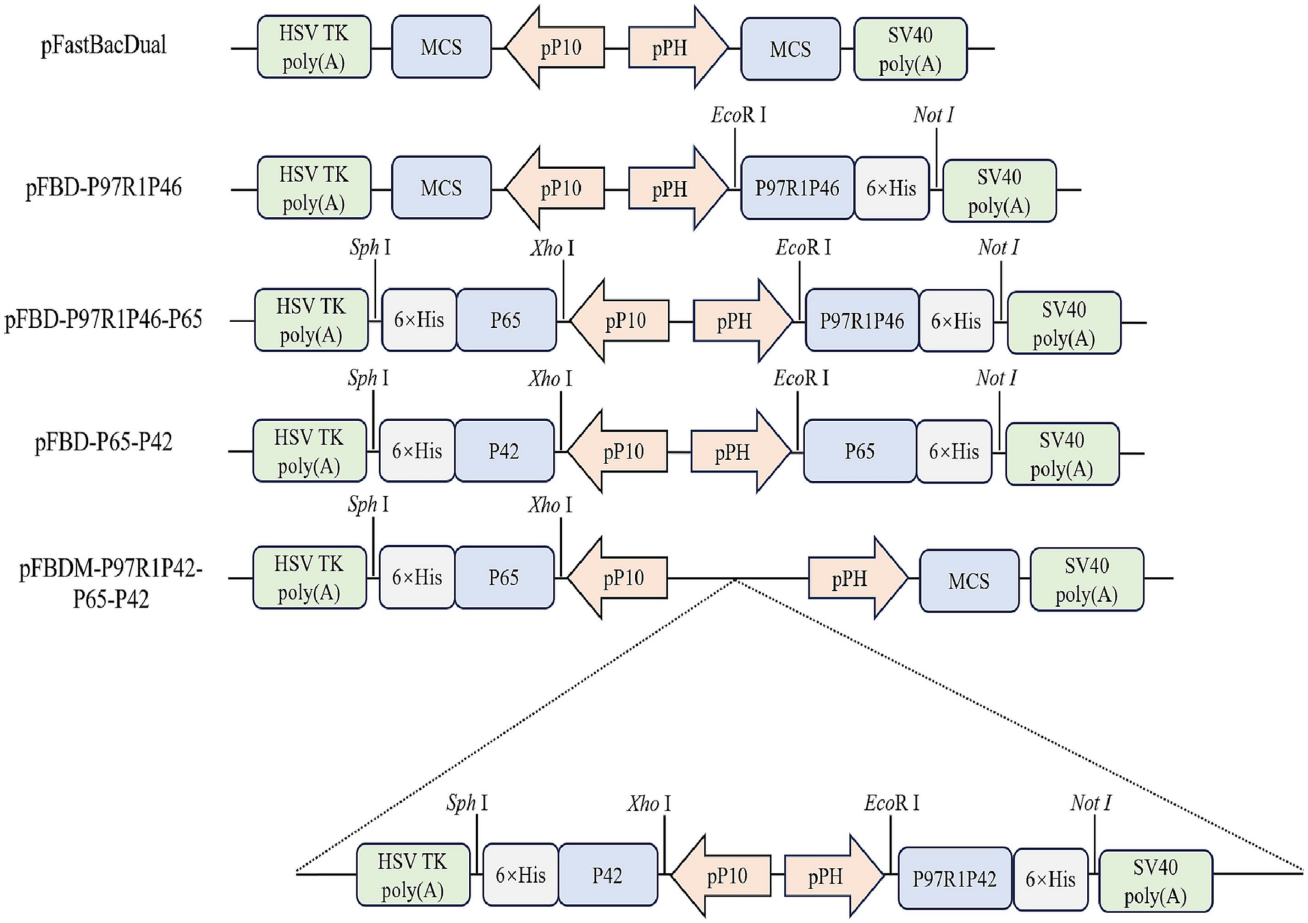
Construction of shuttle plasmids.

The P97 protein plays a significant role in the adhesion process of Mhp, with its R1 region serving as the primary antigenic determinant cluster (21). The P46 protein shows potential as a subunit vaccine. Hence, the R1 region of P97 was concatenated with the P46 protein via a flexible linker peptide (GGSG), resulting in the construct termed P97R1P46. Simultaneously, the P42 and P65 proteins from the Mhp 168 strain were selected as two additional antigenic proteins. The secondary structure of the recombinant proteins was analyzed using the PSIPRED online tool. In this study, we constructed shuttle vectors for the Bac to Bac system: pFBD-P97R1P46, pFBD-P97R1P46-P65, pFBD-P65-P42. Subsequently, the shuttle vector pFBDM-P46P97R1-P65-P42 for the MultiBac system was constructed (Figure 1). After transposition, verification using M13F/R primers confirmed the successful construction of recombinant baculovirus plasmids carrying the target genes.

### 3.2 Identification of recombinant protein

Transfected recombinant baculovirus plasmids into SF9 insect cells during the logarithmic growth phase. After 72 h, observed that the cells ceased to grow, detached from the bottom of the culture dish, and exhibited cell rupture and death. In the control group, SF9 insect cells grew well, adhered normally, maintained clear outlines, and completely covered the bottom of the dish after approximately 72 h. The clarified supernatant collected from the culture represented the first generation of recombinant baculovirus. Subsequent collections from P3 generation recombinant baculoviruses were named sequentially as rAC-P97R1P46, rAC-P97R1P46-P65, rAC-P65-P42, and rAC-P46P97R1-P65-P42.

**Figure 2:**
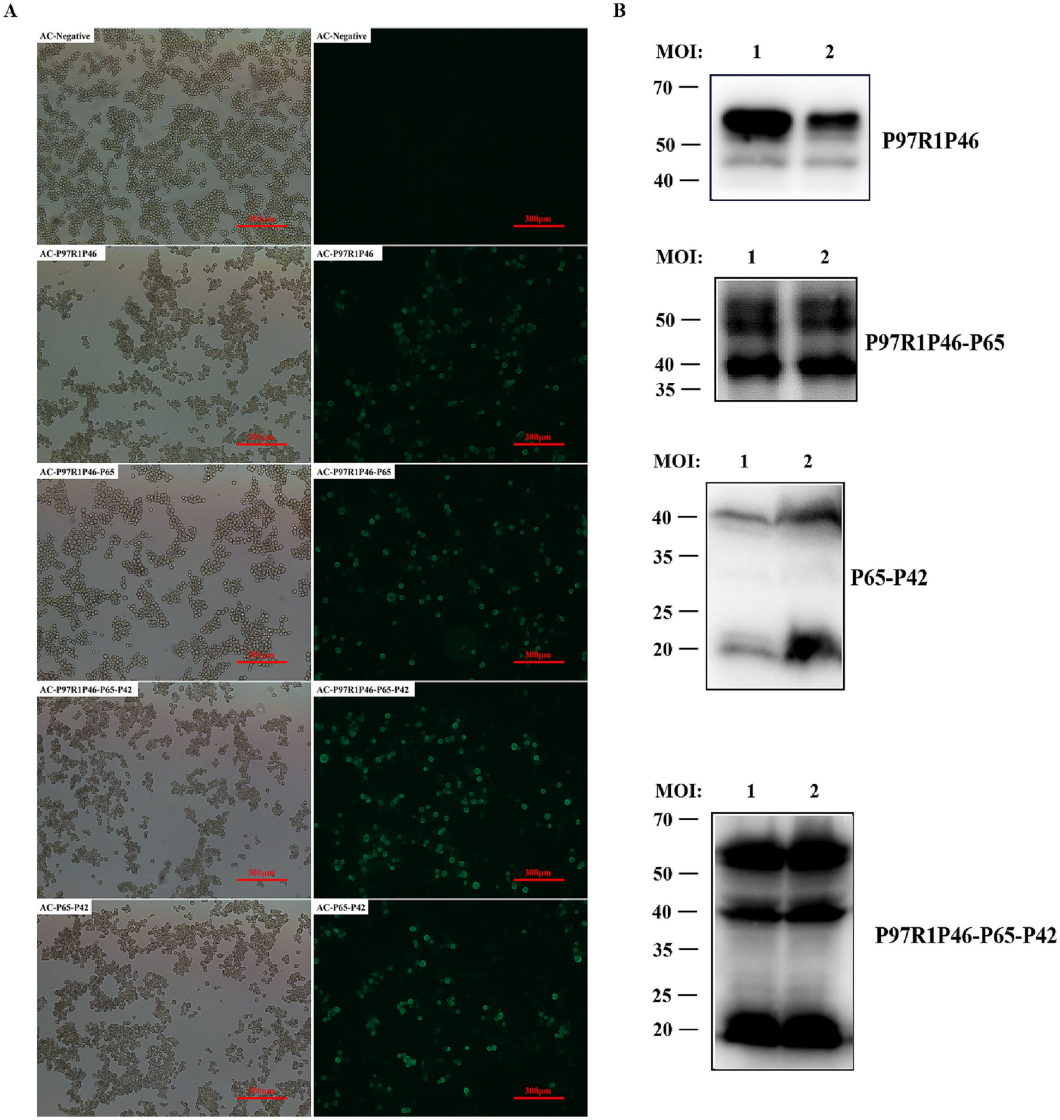
Identification and optimization of recombinant protein expression in baculovirus. (A) Indirect immunofluorescence detection of recombinant baculovirus. (B) Western blot detection of Ac-P97R1P46, Ac-P97R1P46-P65, Ac-P65-P42, and Ac-P97R1P46-P65-P42 after infection with SF9 cells.

After infecting SF9 cells with the recombinant baculovirus mentioned above, the infected cells were lysed and labeled with His-tagged protein antibodies. Through in-direct immunofluorescence analysis, it was observed that all four types of recombinant baculovirus-infected cells emitted green fluorescence (Figure 2A). Using a mouse anti-His tag antibody as the primary antibody and western blotting, specific bands were observed in the cell culture samples (Figure 2B). Samples infected with rAC-P97R1P46 showed specific bands at 46 and 55 kDa. Analysis suggested that the appearance of the 46 kDa band may be due to potential cleavage of the flexible linker (GGSG) connecting P97R1 and P46 during the expression process, with the His-tagged protein located at the C-terminus of the P46 protein. Samples infected with rAC-P97R1P46-P65 exhibited specific bands at 41, 46, and 55 kDa. Samples infected with rAC-P65-P42 showed specific bands at 41 kDa and 20 kDa. Samples infected with rAC-P46P97R1-P65-P42 showed specific bands at 20, 41, 46, and 55 kDa.

### 3.3 Determination of viral valence of recombinant baculovirus

**Figure 3:**
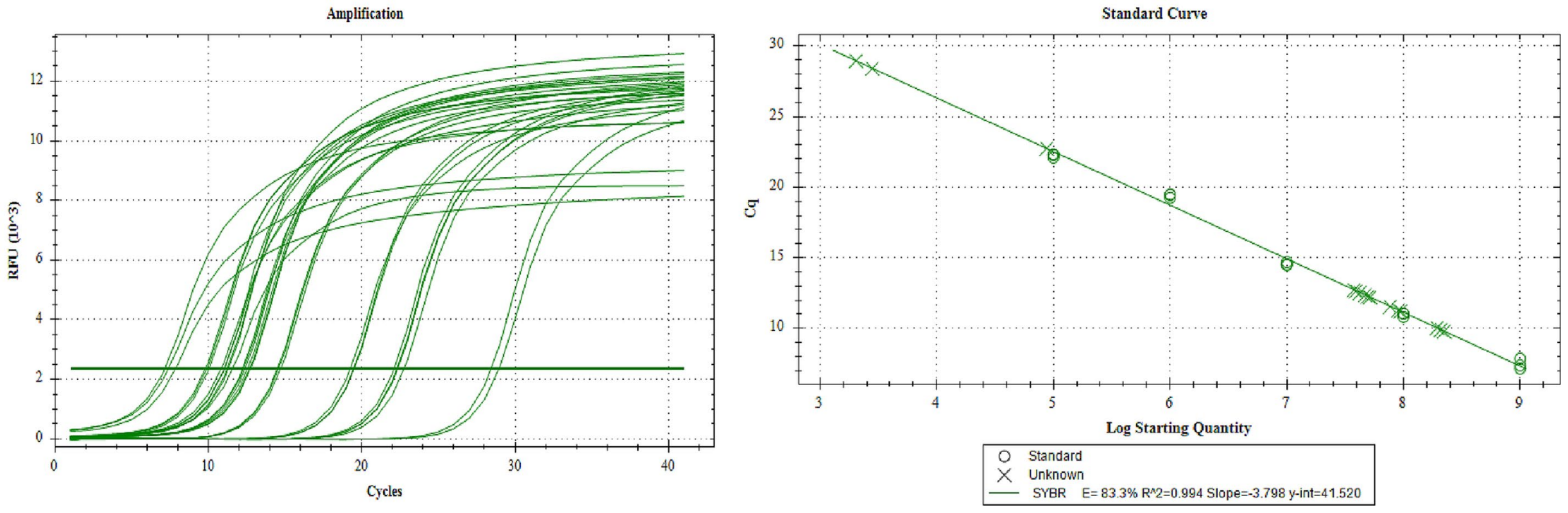
Virulence q-PCR assay of P3 generation recombinant baculovirus.

Collect the P3 generation virus, and determine the copy number of the P3 generation recombinant rod-shaped virus by absolute quantitative PCR. According to the standard curve, the copy numbers of rAC-P97R1P46, rAC-P97R1P46-P65, rAC-P65-P42, and rAC-P46P97R1-P65-P42 are 6.27 × 10^7^ copies/μL, 1.24 × 10^8^ copies/μL, 7.844 × 10^7^ copies/μL, and 1.07 × 10^8^ copies/μL, respectively (Figure 3).

### 3.4 Optimization of recombinant protein expression in baculovirus

**Figure 4:**
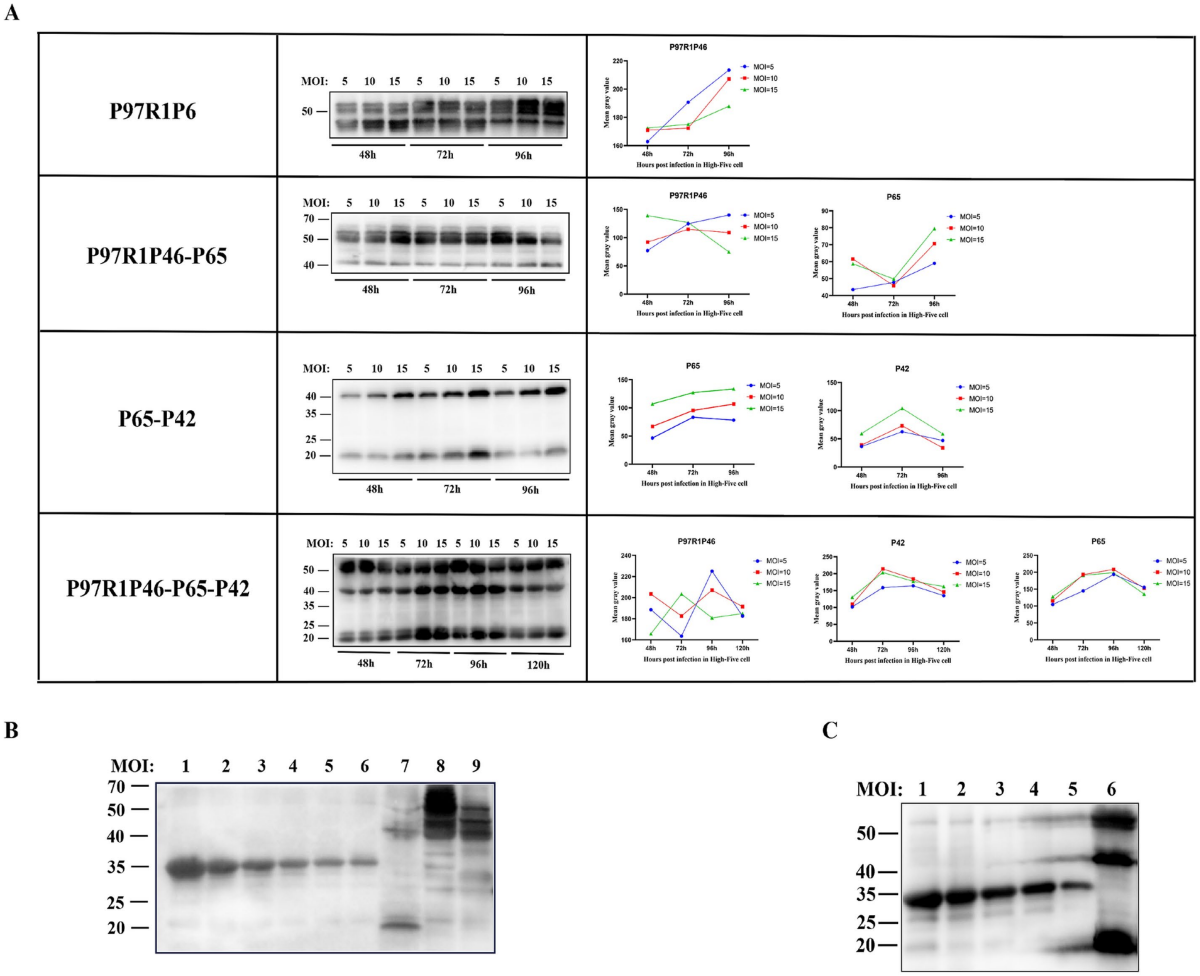
Condition optimization and semi-quantitative analysis of recombinant proteins. (A) Western blot analysis of P97R1P46, P97R1P46-P65, P65-P42, and P97R1P46-P65-P42 proteins under different MOI and time conditions. (B) Semi-quantitative analysis of recombinant proteins. Lanes 1–6 are, in order, 800 μg/mL, 500 μg/mL, 250 μg/mL, 125 μg/mL, 62.5 μg/mL, and 31.25 μg/mL concentrations of standard protein; lane 7 is Ac-P65-P42 infected cell lysate; lane 8 is Ac-P97R1P46 infected cell lysate. Lane 9 is Ac-P97R1P46-P65 infected cell lysate. (C) Semi-quantitative analysis of recombinant proteins. Lanes 1–5 are, in order, 500 μg/mL, 250 μg/mL, 125 μg/mL, 62.5 μg/mL, and 31.25 μg/mL concentrations of standard protein; lane 6 is Ac-P97R1P46-P65-P42 infected cell lysate.

To obtain high yields of recombinant proteins, the P3 generation recombinant baculovirus was used to infect High Five cells, and the conditions of time and MOI were optimized. Cell cultures under various conditions were collected for western blot analysis. The optimal conditions are as follows for each recombinant baculovirus (Figure 4A): rAc-P97R1P46: Inoculated into logarithmic-phase High Five cells with MOI = 10, and cells were harvested at 96 h post-infection. rAc-P97R1P46-P65: inoculated into logarithmic-phase High Five cells with MOI = 5, and cells were harvested at 96 h post-infection. rAc-P65-P42: inoculated into logarithmic-phase High Five cells with MOI = 15, and cells were harvested at 72 h post-infection. rAc-P46P97R1-P65-P42: inoculated into logarithmic-phase High Five cells with MOI = 10, and cells were harvested at 72 h post-infection.

The four recombinant baculoviruses were separately used to infect High Five cells at the logarithmic growth phase under optimal expression conditions, and the proteins were collected. The expression levels of each protein were determined by using a gradient-diluted known concentration of His-tagged protein as the standard. In the cell lysate of cells infected with rAc-P97R1P46, protein bands showed smearing, possibly due to the cleavage of the flexible linker in the recombinant P97R1P46 protein, resulting in breakdown products. The cleavage product, approximately 46 kDa in size, was too close in size and concentration to the recombinant P97R1P46 protein, approximately 55 kDa (Figure 4B). After a four-fold dilution, a semi-quantitative analysis was performed via western blotting, and the results showed clear bands. The protein concentration of the recombinant P97R1P46 protein in the cell lysate of cells infected with rAc-P97R1P46 was approximately 136 μg/mL. In the cell lysate of cells infected with rAc-P65-P42, the protein concentration of the P65 protein was approximately 162 μg/mL, and the concentration of the P42 protein was approximately 207.8 μg/mL in the cell lysate of cells infected with rAc-P97R1P46-P65, the protein concentration of the P97R1P46 protein was approximately 330.6 μg/mL, and the concentration of the P65 protein was approximately 283.4 μg/mL. In the cell lysate of cells infected with rAc-P46P97R1-P65-P42 (Figure 4C), the protein concentration of the P97R1P46 protein was approximately 258 μg/mL, the concentration of the P65 protein was approximately 232.3 μg/mL, and the concentration of the P42 protein was approximately 389.3 μg/mL.

### 3.5 Quality inspection of Mhp subunit vaccines

#### 3.5.1 Physical and chemical properties inspection

The subunit vaccine in this study was prepared using ISA 201 water-in-oil-in-water (W/O/W) adjuvant. The vaccine appears as a milky-white, slightly viscous emulsion with a uniform texture. Using a pipette, a drop of the subunit vaccine was taken and dropped onto the surface of cold water. Part of the drop floated on the water’s surface, forming white droplets, while another part dissolved into the freezing water, creating a mist-like dispersion spreading outward. This observation confirms that the formulation is a water-in-oil-in-water (W/O/W) biphasic emulsion.

#### 3.5.2 Safety inspection

To assess the vaccine’s safety, the prepared subunit vaccine was administered to three healthy Kunming mice at the age of 3 weeks. The results showed that all experimental mice were healthy, survived, and exhibited normal mental status and appetite 28 days after the initial immunization.

Further examination of the clinical signs of the mice in each group revealed no visible swelling, inflammation, or other abnormal reactions caused by the vaccine on the body surface of the experimental mice.

The results above indicate that the subunit vaccine prepared in this study has excellent safety and can be used for subsequent mouse immunization experiments.

### 3.6 Mouse immunization experiments

#### 3.6.1 Immunization experiment

**Figure 5:**
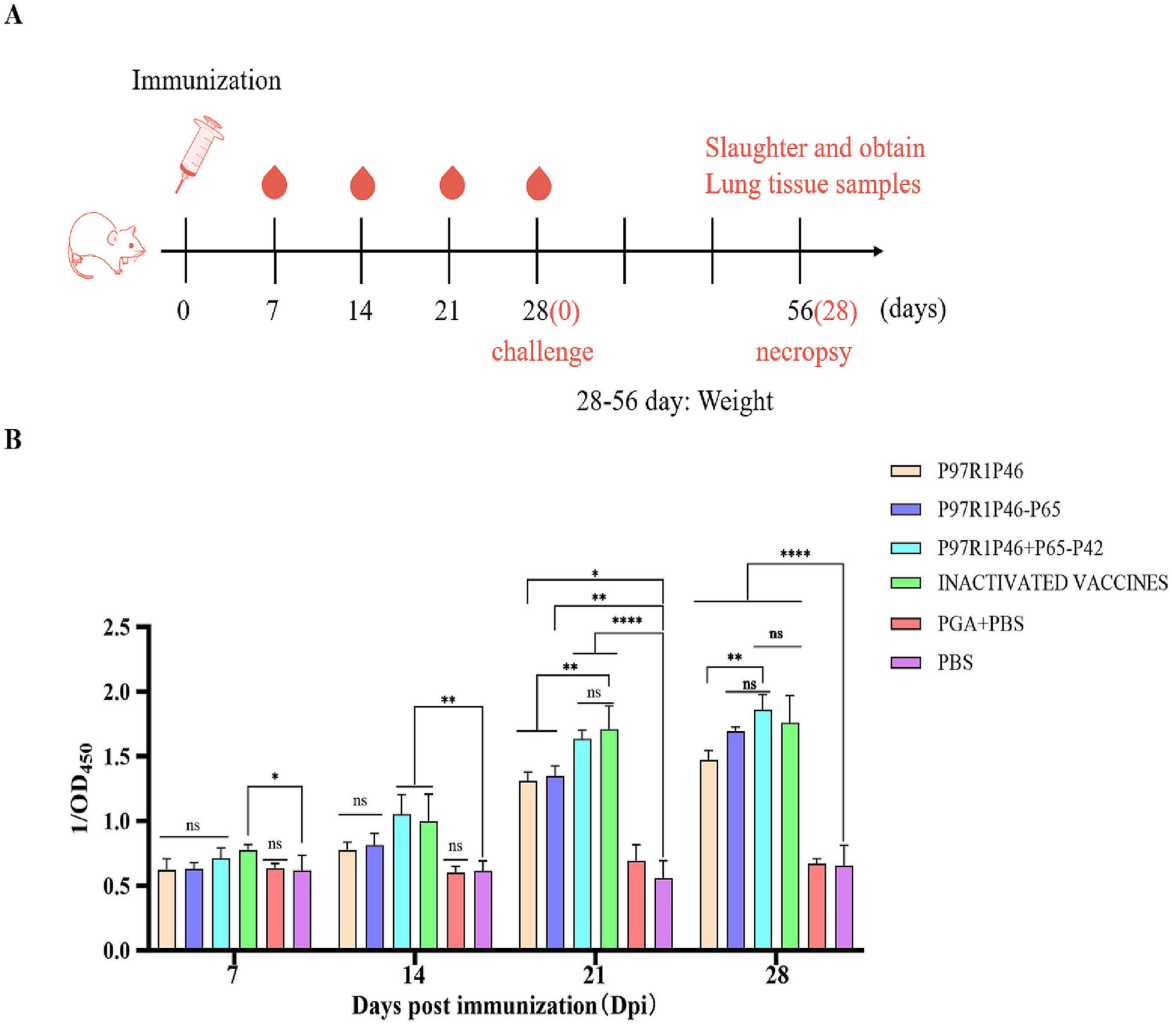
Mouse immunoassay and specific antibody detection. (A) Mouse immunization strategy. (B) The OD_450_ of each group at different days post-immunization (dpi) by Mhp ELISA. Two-way ANOVA: *: *p* < 0.05, **: *p* < 0.01; ns: *p* > 0.05 (no significant difference).

To investigate the immunogenicity of Mhp subunit vaccines prepared with the combinations of P97R1P46, P97R1P46-p65, and P97R1P46 + P65-P42, this study employed ELISA to measure the levels of specific antibodies in the sera of mice at 7, 14, 21, and 28 days after the initial immunization (Figure 5A). The results indicate that the commercialized vaccine group can induce specific antibodies earlier. The antibody levels induced by the P97R1P46 + P65-P42 group are comparable to those induced by the commercialized vaccine group, higher than those induced by the P97R1P46 group and the P97R1P46-P65 group, and significantly different from the PBS group (*p* < 0.05) (Figure 5B). These findings suggest that the Mhp subunit vaccines developed in this experiment all possess good immunogenicity. They can induce the production of specific antibodies in the body after immunization, with the P97R1P46 + P65-P42 group inducing higher levels of specific antibodies.

#### 3.6.2 The detection of IL-2 and IL-4 cytokines in mouse serum was conducted

To investigate the level of cellular immunity in mice after immunization, this experiment employed ELISA to measure the levels of Th1-type (IL-2) and Th2-type (IL-4) cytokines in mouse sera at 28 days after the initial immunization.

**Figure 6:**
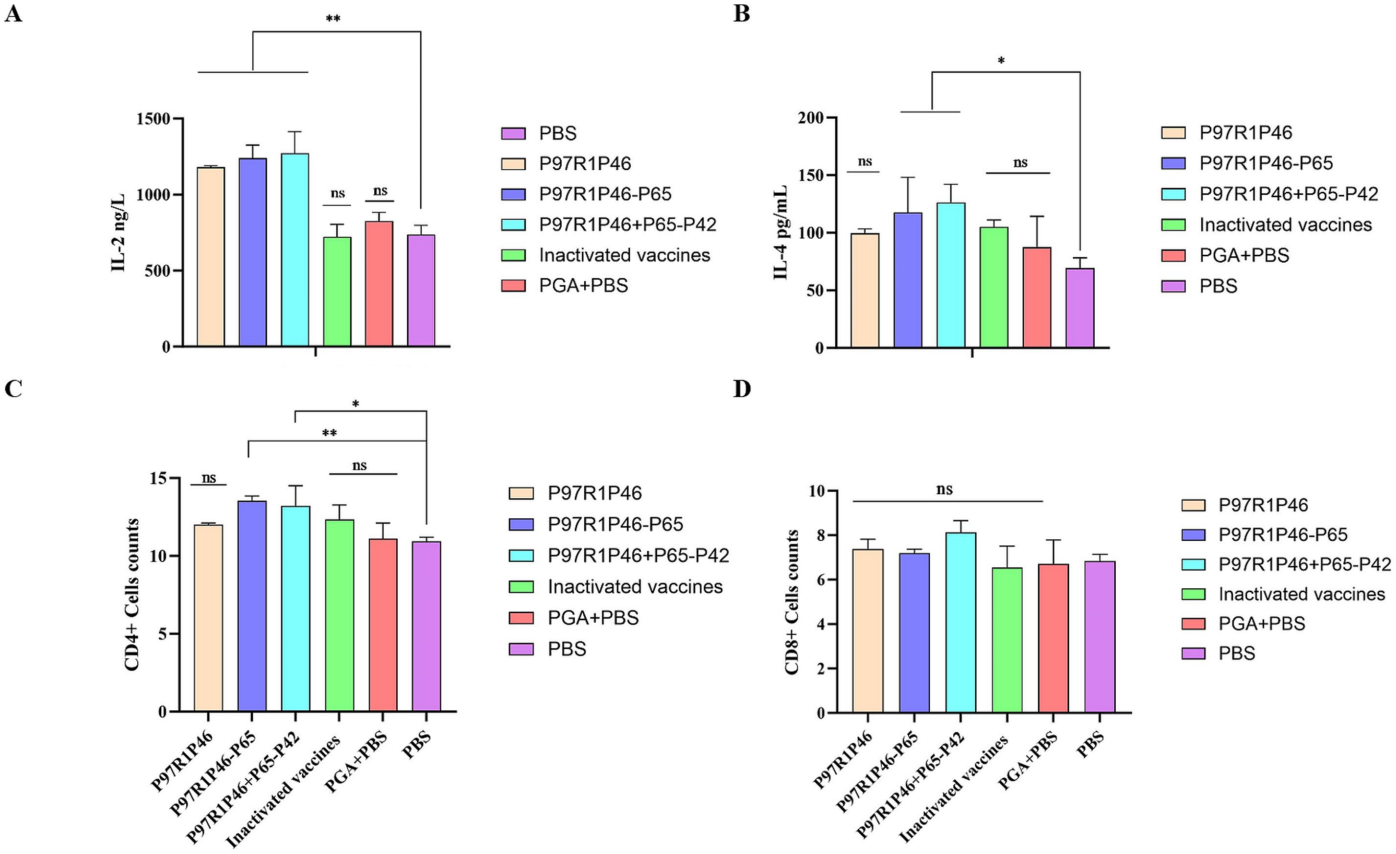
Cytokine assays and immune cell assays in mouse immunoassays. (A) Detection of IL-2 in mouse serum. (B) Detection of IL-4 in mouse serum. (C) CD4^+^ T lymphocyte levels in the spleen of mice. (D) CD8^+^ T lymphocyte levels in the spleen of mice. Two-way ANOVA: *: *p* < 0.05, **: *p* < 0.01; ns: *p* > 0.05 (no significant difference).

The IL-2 secretion levels in the sera of mice from the P97R1P46 + P65-P42 group, the P97R1P46 group, and the P97R1P46-P65 group were significantly higher than those in the PBS group (*p* < 0.01), with the P97R1P46 + P65-P42 group exhibiting the highest levels (Figure 6A). This indicates that the secretion levels of IL-2 in the sera of mice from all subunit vaccine groups were significantly higher than those in the PBS group. The subunit vaccines developed in this study effectively stimulate IL-2 production, thereby inducing a Th1-type immune response. In contrast, the IL-2 levels in the commercial inactivated vaccine group were comparable to those in the PBS group, while the levels in the PGA + PBS group were slightly elevated compared to the PBS group. These results demonstrate that the three subunit vaccines prepared in this study can robustly stimulate IL-2 production in mice, whereas the commercial inactivated vaccine does not provide a positive stimulus for IL-2 production in mice.

The levels of IL-4 secretion in the sera of mice in the P97R1P46 + P65-P42 and P97R1P46-P65 groups were significantly higher than those in the PBS group (*p* < 0.05), with the P97R1P46 + P65-P42 group exhibiting the highest level (Figure 6B). The level of IL-4 secretion in the sera of mice in the P97R1P46 group was higher than that in the PBS group, but the difference was not significant. This indicates that the subunit vaccines prepared in this study can stimulate the production of IL-4 in mice and induce Th2-type immune responses The levels of IL-4 in the commercial inactivated vaccine group and the PGA + PBS group were higher than those in the PBS group, indicating that both the commercial inactivated vaccine and the PGA adjuvant can stimulate the production of IL-4 in mice.

In summary, based on ELISA detection of cytokine secretion levels in mouse sera at 28 days after the initial immunization, it can be concluded that the subunit vaccine prepared in this study can effectively stimulate the production of IL-2 and IL-4 cytokines in mice, inducing Th1-type and Th2-type cellular immune responses, thereby enhancing the cellular immune level of mice. Among them, the subunit vaccine prepared with the combination of P97R1P46 + P65-P42 proteins exhibited the most optimal effect.

#### 3.6.3 Flow cytometric analysis of mouse splenic T lymphocytes

We utilized flow cytometry to investigate the distribution and changes of CD4^+^ and CD8^+^ subsets of T lymphocytes in the spleens of mice from each group. In this study, corresponding fluorescently labeled antibodies APC, FITC, and PE were used to label CD3, CD4, and CD8, respectively. Compensation adjustments were made to eliminate the overlap of fluorescence signals. Box scatter plots were used to describe the distribution of CD4^+^ and CD8^+^ subsets of T lymphocytes in the spleens of mice in each group, and the composition percentages of CD4^+^ and CD8^+^ subsets were compared.

The results indicate that 28 days after the initial immunization, the percentages of CD4^+^ and CD8^+^ lymphocytes in the spleens of mice in each subunit vaccine group were higher compared to the PBS group. Specifically, in terms of CD4^+^ T lymphocytes (Figure 6C), the differences between the P97R1P46-P65 group, and P97R1P46 + P65-P42 group compared to the PBS group were significant (*p* < 0.05), while the P97R1P46 group and the commercial inactivated vaccine group showed a higher level compared to the PBS group, although the difference was not significant. This indicates that P97R1P46-P65 and P97R1P46 + P65-P42 subunit vaccines can positively stimulate helper T lymphocytes. Regarding CD8^+^ T lymphocytes (Figure 6D), the percentages were higher in each subunit vaccine group compared to the PBS group, but the differences were not significant. This suggests that each subunit vaccine group does not stimulate CD8^+^ T lymphocytes. Furthermore, after immunization with subunit vaccines, the ratio of CD4^+^/CD8^+^ lymphocytes in the spleen of mice increased, further indicating that each subunit vaccine effectively enhances the immune response to the organism.

### 3.7 Mouse challenge protection experiment

#### 3.7.1 Histopathological analysis of mouse lung tissue sections after challenge

**Figure 7:**
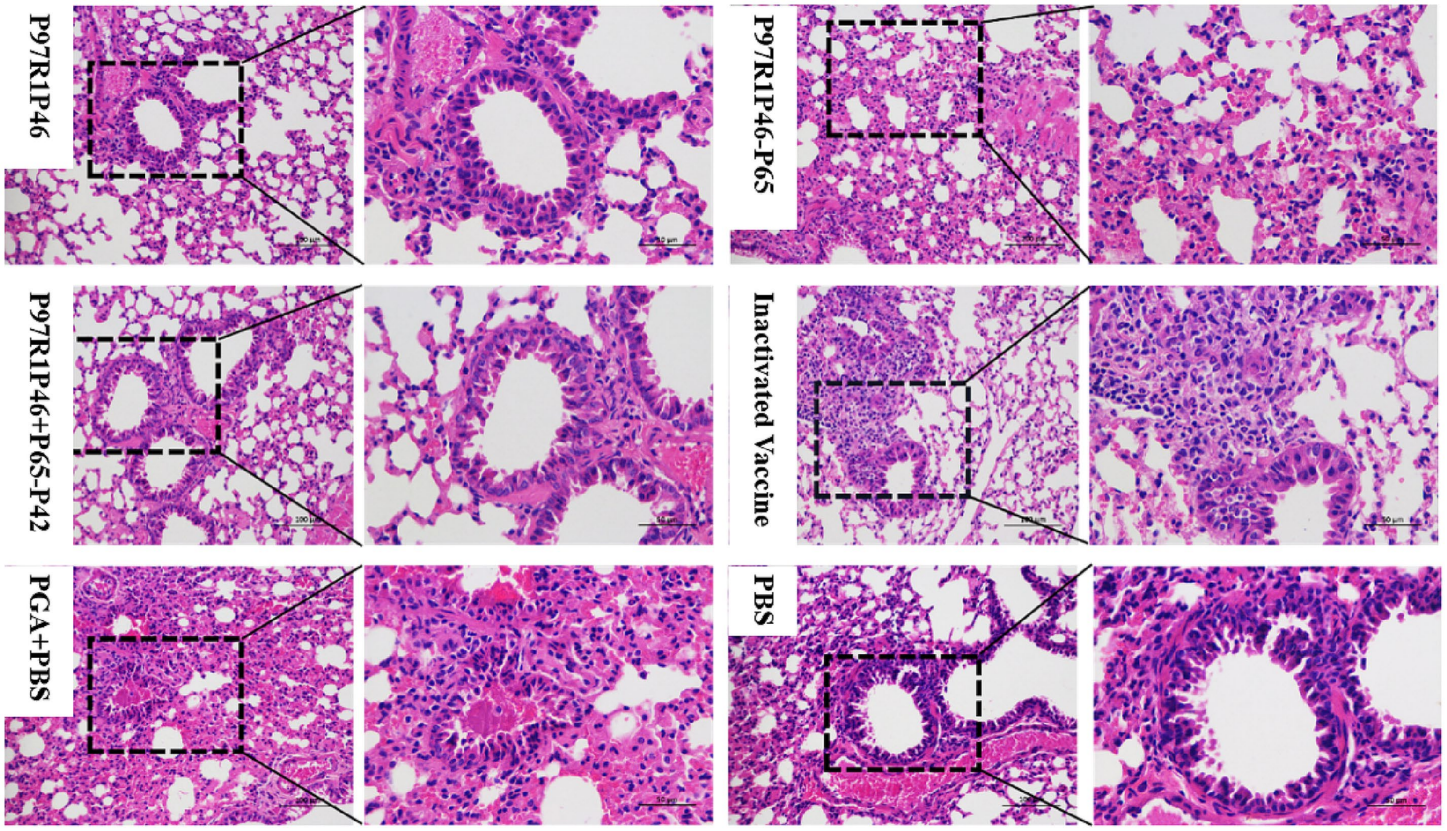
Pathological sections of mouse lungs observed by electron microscopy after Mhp virus challenge.

To analyze the pathological changes in mouse lungs, the isolated lung tissues from each group were fixed in 4% paraformaldehyde, processed by Sevier’s company for paraffin embedding, sectioning, and hematoxylin and eosin staining, and observed under an electron microscope (Figure 7). In the P97R1P46 group, a large number of bronchial epithelial cells were observed to detach, with some of them showing swelling and degeneration, and partial thickening of the alveolar walls with a small amount of exudate. In the P97R1P46-P65 group, there was a significant thickening of the alveolar walls accompanied by inflammatory cell infiltration. In the P97R1P46 + P65-P42 group, no detachment of bronchial epithelial cells was observed, and the alveolar size was uniform, with a small amount of eosinophilic substance exudation in the bronchial lumen. In the commercial inactivated vaccine group, there were uniformly sized alveolar cavities, with a small amount of exfoliation and exudation of alveolar epithelial cells, and no abnormalities in the bronchial lumen were observed. In the PGA + PBS group, a small amount of bronchial epithelial cell detachment was observed, along with exudation of eosinophilic protein-like substances in the bronchial lumen, occasional mild bleeding, significant thickening of alveolar walls resulting in varying alveolar sizes, and slight swelling and degeneration of some bronchial epithelial cells and alveolar epithelial cells. In the PBS group, pathological sections showed thickening of bronchial walls, a small amount of bronchial epithelial cell detachment, a small amount of eosinophilic protein-like substance exudation in the lumen, significant thickening of alveolar walls accompanied by inflammatory cell infiltration, and occasional mild bleeding. Overall, the pathological sections revealed varying degrees of lung damage in each group of mice, with higher levels of pathological damage observed in the PBS and adjuvant groups.

#### 3.7.2 Weight gain of mice after virus challenge

**Figure 8:**
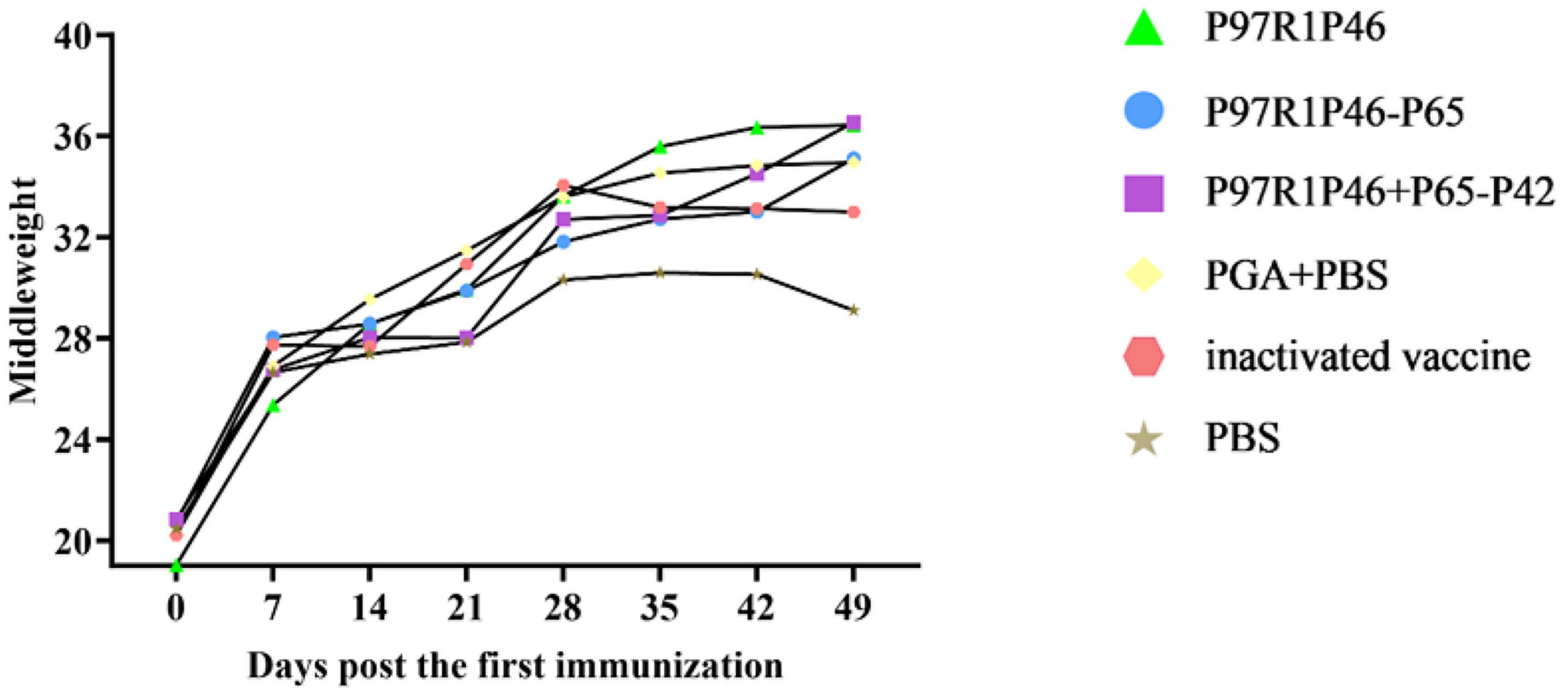
Weight changes in mice after challenge. Changes in body weight in mice after challenge.

Healthy Kunming mice aged 3 weeks were used in this experiment. The weight of mice in each group was measured on the 0th, 7th, 14th, 21st, 28th, 35th, 42nd and 49th day after the first immunization, and the average weight of each group was taken to plot the weight change curve (Figure 8). As shown in the Figure 8, the weight of mice in each group increased significantly up until day 28, the day of challenge. After the 7th day, the mice in each group gradually reached maturity, so the weight gain slowed down, but it could still maintain a steady increase. In this experiment, the Mhp Ji-nan strain freeze-dried tissue virus was used to challenge the mice on the 28th day after the first immunization. After the virus challenge, the appetite of mice in each group decreased to varying degrees, and the daily weight gain slowed down. From the 35th to 49th day, the weight gain of mice in the PBS group and the adjuvant group stagnated or even decreased, while the mice in the P97R1P46-P65 group and the P97R1P46 + P65-P42 group still maintained weight gain. In summary, mouse experiments showed that the combination protein of P97R1P46 + P65-P42 had better immunogenicity than other subunit vaccines, thus subsequently, the genes P97R1P46, P65, and P42 were simultaneously cloned into the pFastMultibacDual vector for co-expression using the MultiBac system, followed by piglet immunization experiments.

### 3.8 Piglet immunization experiments

#### 3.8.1 Serum-specific antibody level ELISA detection

**Figure 9:**
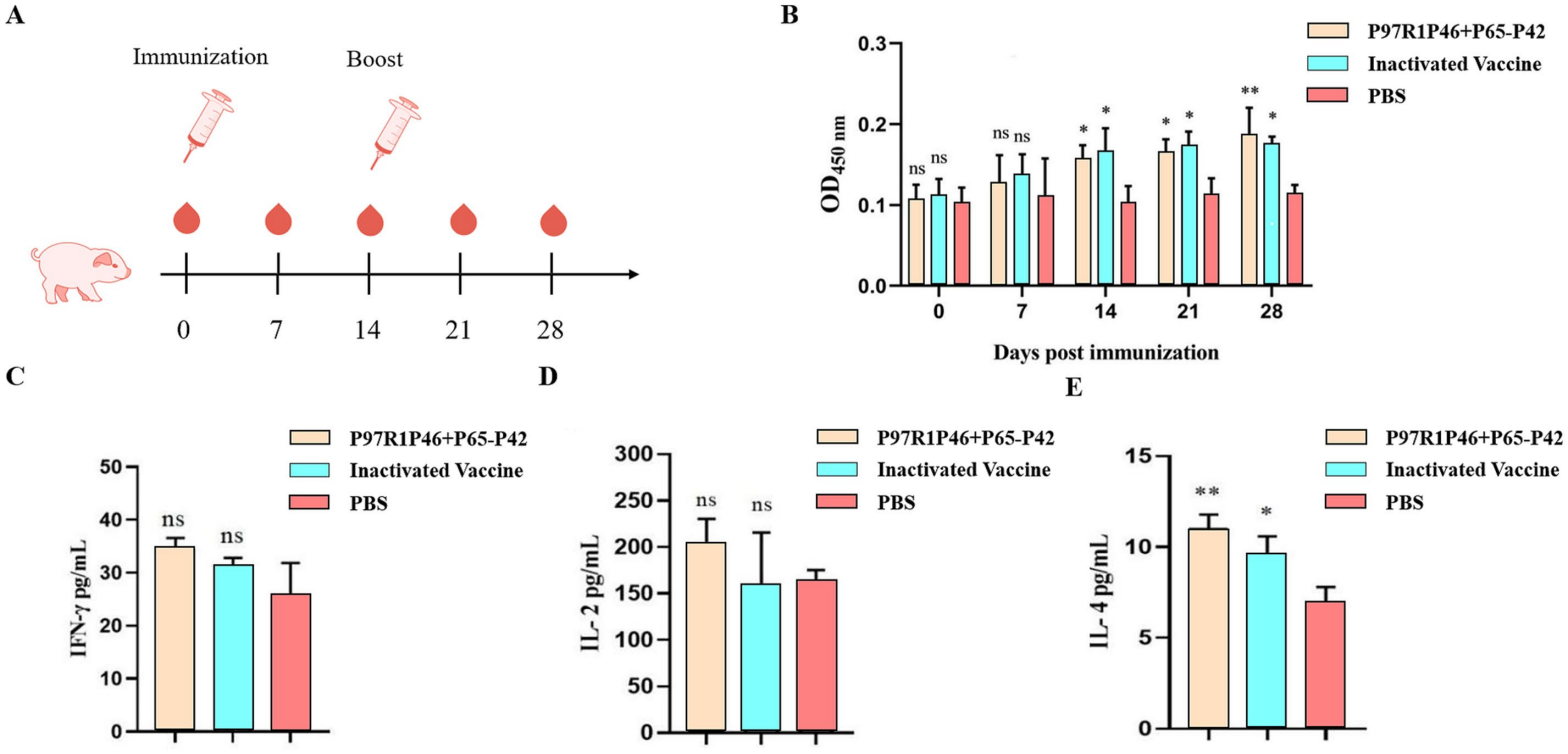
Piglet immunization experiments. (A) Piglet immunization strategy. (B) The OD_450_ of each group at different days post-immunization (dpi) by Mhp ELISA. (C) ELISA was used to determine the con-tents of IFN-γ cytokines in the serum of pigs 28 days after the first immunization. (D) ELISA was used to determine the contents of IL-2 cytokines in the serum of pigs 28 days after the first immunization. (E) ELISA was used to determine the contents of IL-4 cytokines in the serum of pigs 28 days after the first immunization. Two-way ANOVA: *: *p* < 0.05, **: *p* < 0.01; ns: *p* > 0.05 (no significant difference).

The recombinant protein P46P97R1-P65-P42 and ISA 201 VG (W/O/W) adjuvant were mixed at a 1:1 ratio to prepare the Mhp subunit vaccine. This vaccine, along with the commercial inactivated Mhp vaccine, was immunized into 1-month-old piglets, without Mhp antigen and antibody detection, at 0 day and 14 days (Figure 9A). To investigate the immune effect of the Mhp subunit vaccine on piglets, serum samples were collected from each experimental group piglet at 0 day, 7 days, 14 days, 21 days, and 28 days, and the Mhp antibody levels were detected using an indirect ELISA method, with the results represented by the OD_450_ of the test serum (Figure 9B).

At 7 days post-primary immunization, the levels of Mhp-specific antibodies in the serum of piglets from the P46P97R1-P65-P42 group and the commercial inactivated Mhp vaccine group showed a slow increase over time compared to the PBS group, but the difference was not significant (*p* > 0.05). At 14 days post-primary immunization, the antibody levels in all vaccine groups were significantly higher than those in the PBS group (*p* < 0.05), with the commercial inactivated Mhp vaccine group showing higher levels than the P46P97R1-P65-P42 group. At 28 days post-primary immunization, the levels of Mhp-specific antibodies in the serum of piglets from the P46P97R1-P65-P42 group were significantly higher than those in the PBS group (*p* < 0.01) and higher than those in the commercial inactivated Mhp vaccine group. These results indicate that from day 0 to day 21 post-primary immunization, the levels of Mhp-specific antibodies induced by P46P97R1-P65-P42 were lower than those induced by the commercial inactivated Mhp vaccine. However, after 14 days post primary immunization, the antibody levels induced by the commercial inactivated Mhp vaccine were higher than those induced by the P46P97R1-P65-P42 subunit vaccine. Thus, the commercial inactivated Mhp vaccine can induce a high and significant level of antibodies in a shorter period after the first immunization compared to the Mhp subunit vaccine. However, by day 28 the P46P97R1-P65-P42 subunit vaccine stimulated higher levels of Mhp-specific anti-bodies after the booster than the commercial Mhp vaccine.

#### 3.8.2 The detection of IL-2, IFN-γ, and IL-4 cytokines in piglet serum was conducted

To investigate the cellular immune response post-vaccination in experimental pigs, this study employed ELISA to measure the levels of IFN-γ, IL-2, and IL-4 cytokines in the serum of pigs at 28 days after the initial vaccination. This was done to study the levels of Th1-type (IFN-γ and IL-2) and Th2-type (IL-4) cellular immune response in the vaccinated pigs.

The results of IFN-γ detection are as follows (Figure 9C): at 28 days after the initial vaccination, the levels of IFN-γ in the serum of experimental pigs from each vaccinated group were slightly higher than those in the PBS group, but the difference was not significant (*p* > 0.05). Among them, the levels of IFN-γ in the serum of pigs vaccinated with the Mhp subunit vaccine were slightly higher than those vaccinated with the commercial inactivated Mhp vaccine.

The results of IL-2 detection are as follows (Figure 9D): compared to the PBS group, the serum IL-2 levels in the Mhp subunit vaccine group were slightly elevated, but the difference was not significant (*p* > 0.05). The IL-2 levels in the commercial in-activated Mhp vaccine group were comparable to those in the PBS group.

The results of IL-4 detection are as follows (Figure 9E): compared to the PBS group, the serum IL-4 levels in the Mhp subunit vaccine group showed a significant increase (*p* < 0.01), while the IL-4 levels in the commercial inactivated Mhp vaccine group were higher than those in the PBS group, with a relatively significant difference (*p* < 0.05). Additionally, the Mhp subunit vaccine group exhibited higher IL-4 levels compared to the commercial inactivated Mhp vaccine group.

The results demonstrate that the Mhp subunit vaccine developed in this study significantly elevates IL-4 cytokine levels in the peripheral blood of immunized pigs. This finding suggests that P97R1P46-P65-P42 effectively induces a robust humoral immune response in piglets but fails to elicit a substantial cellular immune response.

## 4 Discussion

*Mycoplasma hyopneumoniae* (Mhp) poses a significant threat to the global swine industry due to its role as a respiratory pathogen. Vaccination remains the most effective strategy for preventing and controlling Mhp infections in pigs, making the development of safe and effective vaccines crucial for mitigating the impacts of porcine mycoplasma pneumonia.

The efficacy of subunit vaccines is heavily influenced by antigen selection. The R1 region of the P97 protein independently facilitates the adhesion process, and Mhp’s adhesion involves multiple adhesins—over 60 identified to date (22–24). Studies indicate that focusing solely on a single antigen protein is insufficient; a vaccine based only on P97 does not confer adequate immune protection in pigs (10, 25). Membrane proteins P46 and P65 are among the most studied for subunit vaccine development (25, 26). P42, a heat shock protein, has been demonstrated to inhibit Mhp growth (27). By linking the R1 region of P97 to P46 using a flexible linker (GGSG), we created the P97R1P46 fusion protein, which was co-expressed with two other proteins in various combinations.

Utilizing an insect cell expression system, known for high expression levels and post-translational modifications, we optimized the nucleotide sequence for insect codons to ensure effective expression. Through transposition and transfection in SF9 cells, we obtained the P3 generation recombinant baculoviruses and expressed the protein combinations (P97R1P46, P97R1P46-P65, P97R1P46 + P65-P42) in High Five cells. These were formulated into subunit vaccines and used to immunize mice in challenge experiments. Results showed that while the commercial vaccine induced specific antibodies most rapidly, the P97R1P46 + P65-P42 group induced comparable antibody levels 7 days post-immunization, slightly surpassing the commercial vaccine at 28 days. Cytokine assays in mouse blood revealed that the P97R1P46 + P65-P42 group significantly induced IL-2 and IL-4 production compared to controls, and flow cytometry confirmed higher CD4^+^ T cell content in this group. These findings suggest that our P97R1P46 + P65-P42 subunit vaccine induces both humoral and cellular immunity in mice. Subsequent challenge experiments further demonstrated superior protective efficacy in this group, validating the multi-antigen approach over single-antigen strategies.

Expressing these three proteins together to create the P97R1P46-P65-P42 subunit vaccine for piglets induced high levels of specific antibodies and IL-4. However, IL-2 and IFN-γ levels did not significantly differ from controls, suggesting the vaccine primarily elicited humoral rather than cellular immunity. This might be due to inefficient antigen presentation to T cells or insufficient antigen content to trigger a strong T cell response, resulting in lower IFN-γ and IL-2 production. Why were different cytokine trends observed in mouse and piglet experiments? We hypothesize that this discrepancy arises from the distinct functionalities of the innate and adaptive immune systems in mice and pigs (28). The immune responses of mice and pigs to foreign antigens vary significantly. Moreover, Th1 and Th2 cells can mutually regulate and restrict each other’s functions through the cytokines they secrete. For instance, Th1 cells secrete IFN-γ, which inhibits the proliferation of Th2 cells, whereas Th2 cells secrete IL-10, which suppresses the function of Th1 cells (29). The results also confirm that vaccines should rather be tested in the target host to obtain true results. Future work should focus on enhancing cellular immunity induced by this subunit vaccine. Virus-like particles (VLPs) vaccines could be a potential solution, as they effectively mimic natural virus structures and sizes, with antigens fully displayed on their surfaces for better presentation to T cells (30, 31). VLPs can also act as adjuvants, enhancing immune responses through dendritic cell activation and maturation, critical for initiating T cell responses (32, 33). Nevertheless, our P97R1P46-P65-P42 subunit vaccine successfully induced high levels of humoral immunity, and the combination of these four antigen proteins represents a promising approach for Mhp vaccination.

## 5 Conclusion

In summary, this study utilized two different insect cell expression systems, namely the Bac to Bac system and the MultiBac system. Initially, proteins prepared using the Bac to Bac system were combined with PGA adjuvant in various combinations (P97R1P46, P97R1P46-P65, and P97R1P46 + P65-P42) to form subunit vaccines. Following mouse immunization and challenge experiments, the combination yielding the best immune response was identified as P97R1P46 + P65-P42. Subsequently, these three proteins were co-expressed using the MultiBac system, and the expressed proteins were mixed with PGA adjuvant to produce the subunit vaccine P97R1P46-P65-P42. Immunization experiments in pigs demonstrated that this vaccine could induce a high level of humoral immunity. By incorporating four antigenic proteins, this subunit vaccine addressed the issue of poor efficacy observed with single-antigen subunit vaccines against *Mycoplasma hyopneumoniae*, providing insights for the development of novel Mhp vaccines.

## Data Availability

The raw data supporting the conclusions of this article will be made available by the authors, without undue reservation.
