# Supplementary figures and images for: A subunit vaccine based on P97R1, P46, P42, and P65 from Mycoplasma hyopneumoniae can induce significant immune response in piglets
Source: Front Vet Sci. 2024 Nov 13;11:1493650. doi: 10.3389/fvets.2024.1493650 (PMC11599267; doi:10.3389/fvets.2024.1493650)

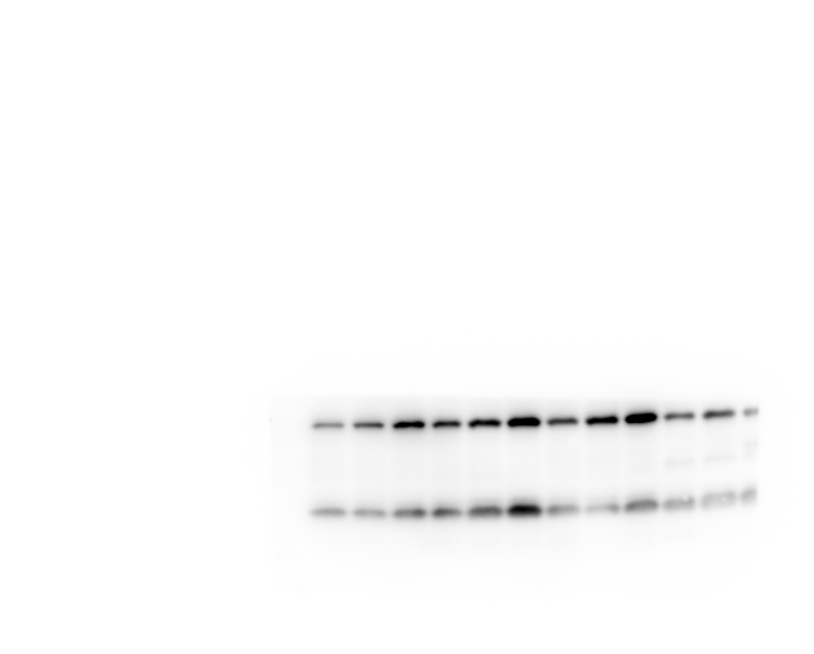

Supplement: Supplementary file 1 [file Data_Sheet_1.zip › original image/Optimization of expression conditions of P65-P42 protein.tif]

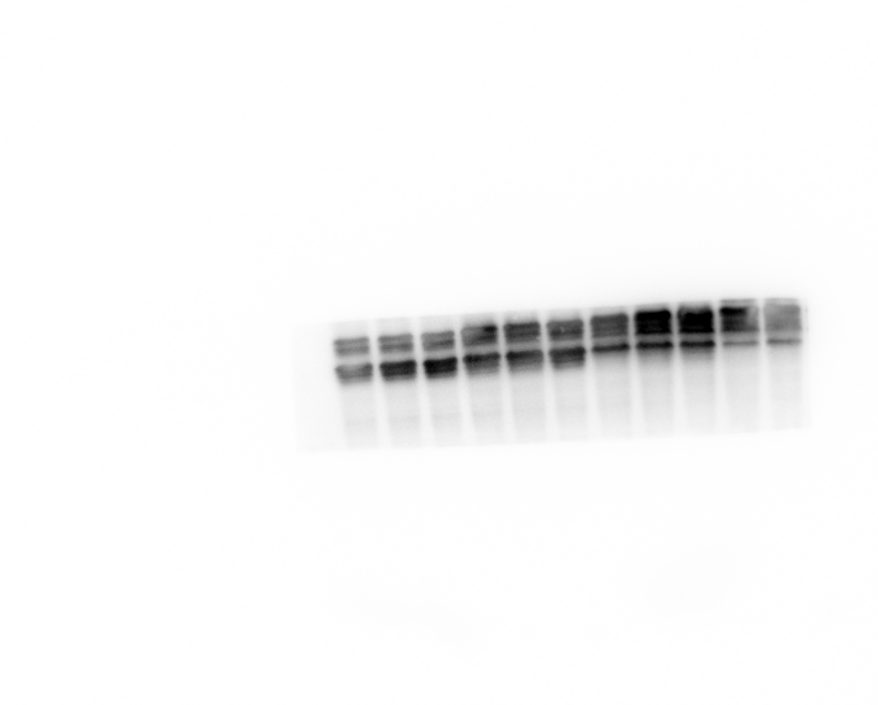

Supplement: Supplementary file 1 [file Data_Sheet_1.zip › original image/Optimization of expression conditions of P97R1P46 protein.tif]

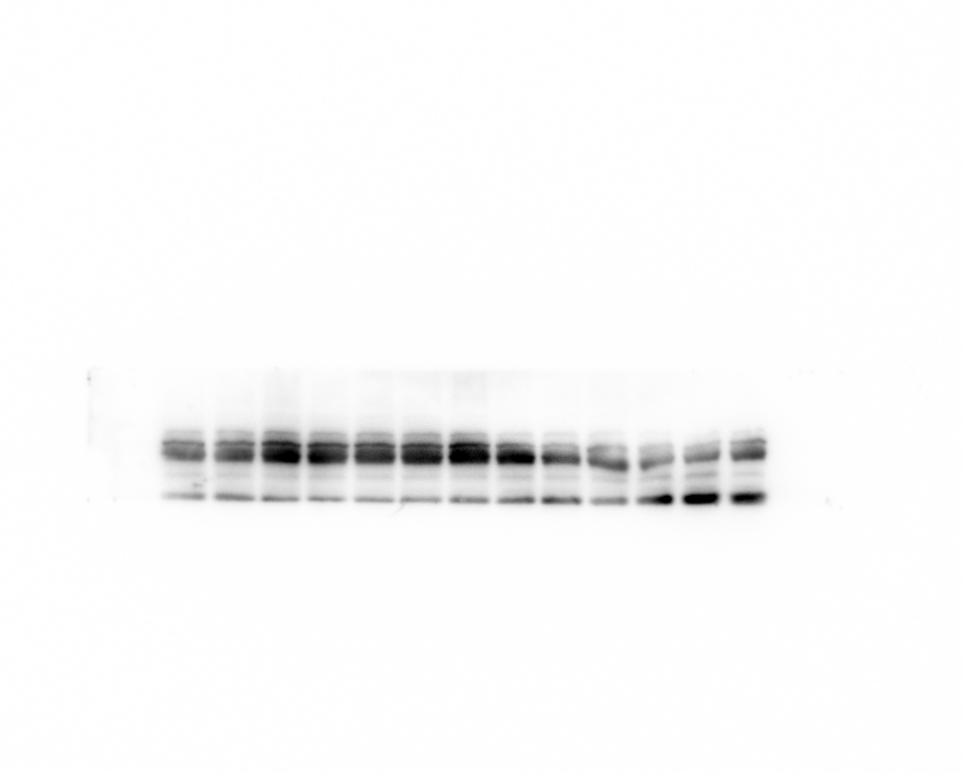

Supplement: Supplementary file 1 [file Data_Sheet_1.zip › original image/Optimization of expression conditions of P97R1P46-P65 protein.tif]

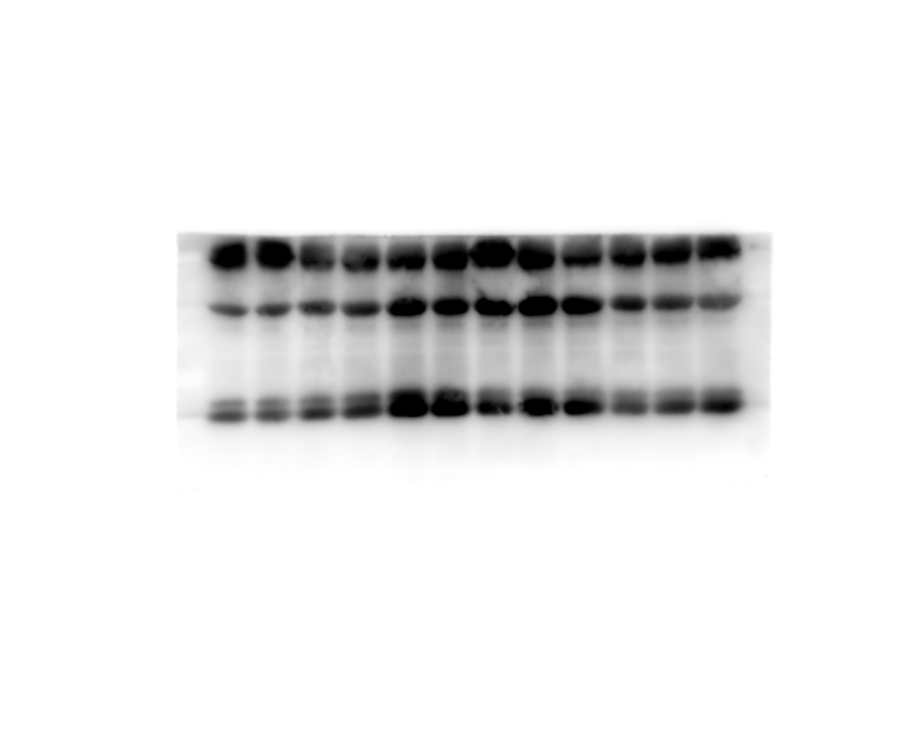

Supplement: Supplementary file 1 [file Data_Sheet_1.zip › original image/Optimization of expression conditions of P97R1P46-P65-P42 protein.tif]

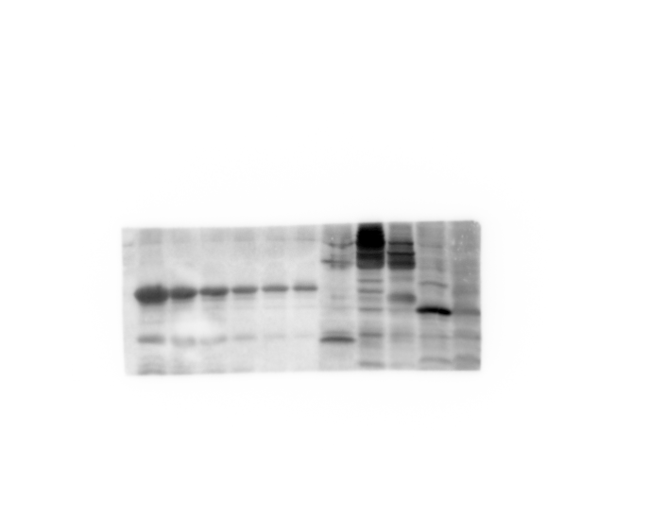

Supplement: Supplementary file 1 [file Data_Sheet_1.zip › original image/Semi-quantitative analysis of P65-P42, P97R1P46, P97R1P46-P65.tif]

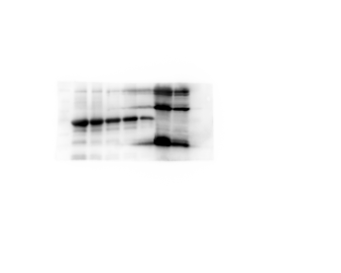

Supplement: Supplementary file 1 [file Data_Sheet_1.zip › original image/Semi-quantitative analysis of P97R1P46-P65-P42.tif]

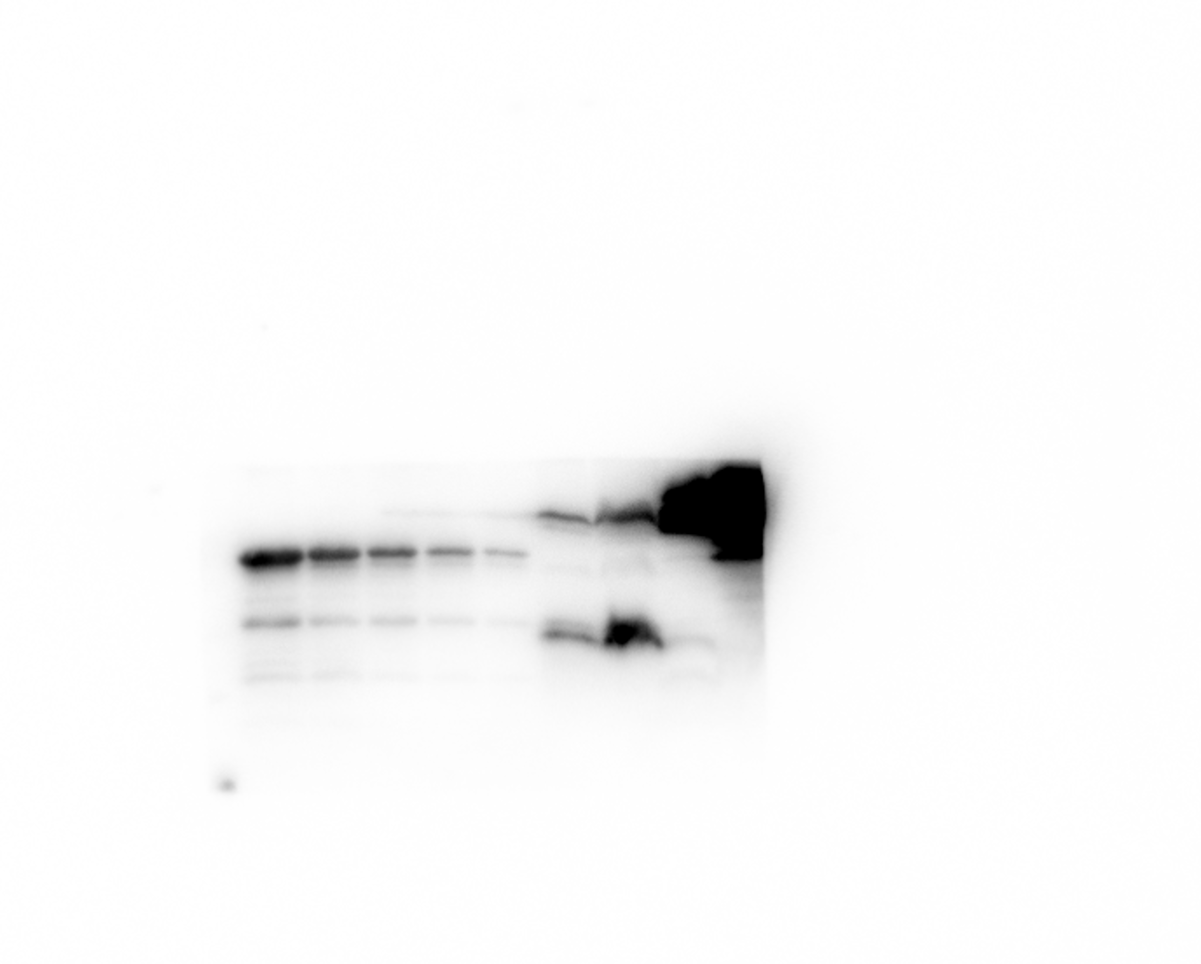

Supplement: Supplementary file 1 [file Data_Sheet_1.zip › original image/Western blot identification of P65-P42 protein..tif]

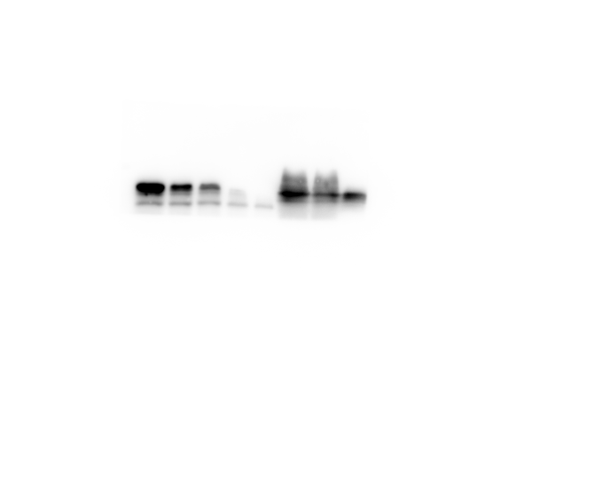

Supplement: Supplementary file 1 [file Data_Sheet_1.zip › original image/Western blot identification of P97R1P46 protein..tif]

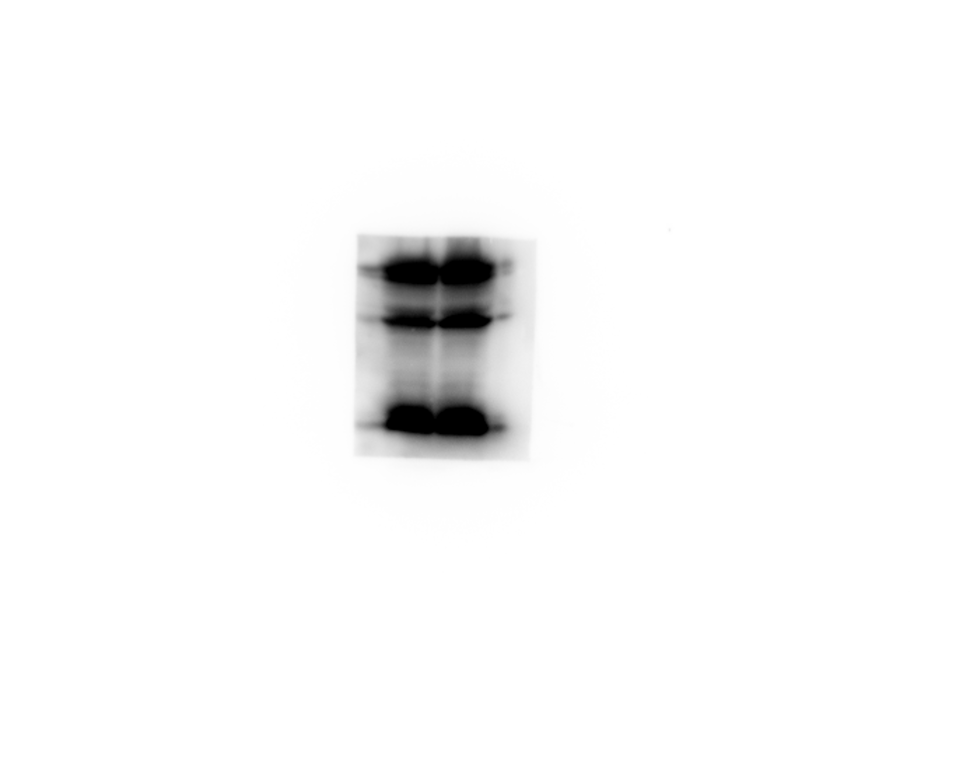

Supplement: Supplementary file 1 [file Data_Sheet_1.zip › original image/Western blot identification of P97R1P46-P65-P42 protein..tif]

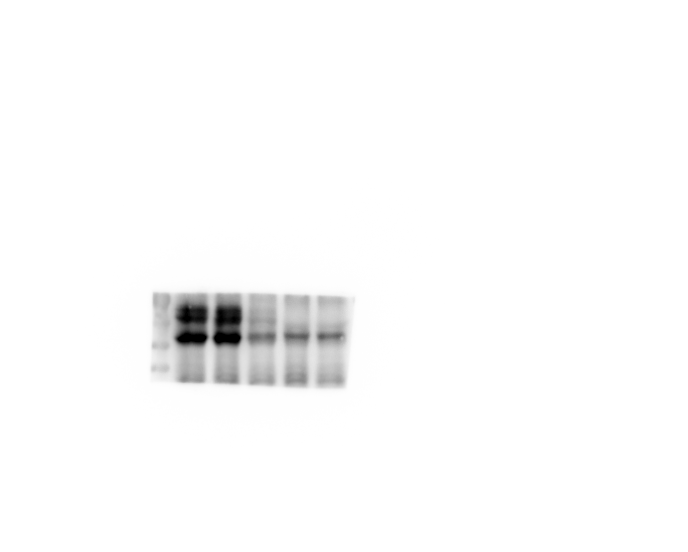

Supplement: Supplementary file 1 [file Data_Sheet_1.zip › original image/Western blot identification of P97R1P46-P65protein. .tif]
